# Data on draft genome sequence of *Bacillus* sp. strain MHSD28, a bacterial endophyte isolated from *Dicoma anomala*

**DOI:** 10.1016/j.dib.2019.104524

**Published:** 2019-09-19

**Authors:** Sephokoane Cindy Makuwa, Mahloro Hope Serepa-Dlamini

**Affiliations:** Department of Biotechnology and Food Technology, Faculty of Science, University of Johannesburg, Doornfontein Campus, PO Box 17011 Doornfontein 2028, Johannesburg, South Africa

**Keywords:** *Bacillus*, *Dicoma anomala*, Bacterial endophyte, Phylogenomic analysis

## Abstract

Here, we present the draft genome sequence of *Bacillus* sp. strain MHSD28 which was sequenced, and assembled with a total length of 5,571,729 bp. The genome has 43 contigs, the largest contig with 1,785,042 bp, N_50_ of 1,474,247 bp, G + C% content of 35.23%. The strain was isolated from surface sterilized leaves of *Dicoma anomala*, obtained in Limpopo province, South Africa. The genome has 5792 total genes which include 5701 protein coding sequences (CDS), 192 pseudogenes, 7 rRNA genes with 3 operons (5S, 16S and 23S), 79 tRNA genes and 5 noncoding RNA (ncRNA) genes. This whole genome shotgun project has been deposited in DDBJ/ENA/GenBank under accession number VHIV00000000. The version described in this paper is version VHIV01000000.

**Table:** Specifications Table

Subject area	Biology
More specific subject area	Bacterial Genomics, Bioinformatics, Molecular biology, Plant microbiology
Type of data	Tables, Figures
How data was acquired	Genome sequencing with Illumina MiSeq at Agricultural Research Council (ARC), Onderstepoort, South Africa. Genome assembly with *de novo* assembly Unicycler (Galaxy version 0.4.6.0). Genome annotation with NCBI Genome Automatic Annotation Pipeline (PGAAP)
Data format	Assembled and Analyzed
Experimental factors	Genomic DNA extraction, genome assembly and annotation.
Experimental features	Genomic DNA extraction was performed with Zymo Research Fungal/Bacterial DNA MiniPrep Kit (Catalog no: D6005). Whole genome Sequencing of *Bacillus* sp. strain MHSD28 was sequenced with Illumina MiSeq platform at Agricultural Research Council (ARC), Onderstepoort, South Africa. The genome was assembled with *de novo* assembly Unicycler (version 0.4.6.0) a web platform on Galaxy (https://usegalaxy.org).Quast (Galaxy version 0.4.6.3) was used to assess genome quality which was annotated with NCBI Prokaryotic Genome Automatic Annotation Pipeline (PGAAP).
Data source location	*Bacillus* sp. strain MHSD28 was isolated from fresh sterilized leaves of medicinal plant *Dicoma anomala* obtained in Limpopo province, South Africa.
Data accessibility	Genome assembly and annotation data are found in this article. All raw, assembled and annotation data have been deposited in DDBJ/ENA/GenBank with BioProject number: PRJNA549839, BioSample number: SAMN12098152 under the accession VHIV00000000 (https://www.ncbi.nlm.nih.gov/nuccore/VHIV00000000). The version described in this paper is version VHIV01000000.

**Table:** 

**Value of the data** • This study will identify genes, important for bacterial endophyte lifestyle. • The outcome of whole genome sequence of *Bacillus* sp. strain MHSD28 will improve data analysis in genomics for studies of plants associated with *Bacillus* species. • Genome sequence analysis of *Bacillus* sp. strain MHSD28 will provide further information to distinguish the differences between strains within the genus *Bacillus* at gene level.

## 1 Data

Plant growth promoting bacteria (PGPB) are microorganisms that stimulate plant growth and suppress plant diseases. Bacterial strains that have been successfully utilized as PGPB include species from genera *Bacillus*, *Pseudomonas* and *Stenotrophomonas* [1]. *Bacillus* is a genus that belongs to the phylum Firmicutes, with diverse bacterial species that are Gram-positive, rod-shaped and spore formers [2]. *Bacillus* species are ubiquitous in nature and have been isolated from numerous environments such as plants, animals, freshwater and the soil [3]. Some strains of *Bacillus* genus promote growth of different plants through various mechanisms, such as biofertilization, increasing accessibility of primary nutrients such as nitrogen, phosphate, potassium for the plant, phytostimulation through the production of phytohormones such as indole acetic acid (IAA), auxin and ethylene, as well as biocontrol by production of antimicrobial metabolites [4], [5], [6]. In addition, *Bacillus* species can form spores, an advantage that allows this group of bacteria to survive in unfavorable conditions [7].

*Bacillus* sp. strain MHSD28 was isolated from surface sterilized leaves of *Dicoma anomala*, and initially identified using the 16S rRNA gene (GenBank accession number MN029053). *D. anomala* is a medicinal plant with various pharmacological properties such as anti-inflammatory, anti-bacterial, anti-plasmodial, anti-helminthic, anti-viral, analgesic and wound healing activities [8]. The plant was isolated from Limpopo province, South Africa. The genome sequence of *Bacillus* sp. strain MHSD28 was sequenced with Illumina MiSeq platform. *De novo* assembly was performed on Galaxy web platform (https://usegalaxy.org) using Unicycler (version 0.4.6.0) and assessed with Quast (version 0.4.6.3). Genes were predicted using the NCBI Prokaryotic Genome Automatic Annotation Pipeline (PGAAP) [9]. The genome annotation statistics are provided in Table 1. The resulting draft genome of *Bacillus* sp. strain MHSD28 has 5,571,729 bp, with 43 contigs, the largest contig of 1,785,042 bp, N_50_ of 1,474,247 bp and G + C% content of 35.23%. *Bacillus* is a distinctive genus with G + C% content ranging from 34 to 35% (*Bacillus cereus* and other *Bacillus* related species) to 44–46% (*Bacillus subtilis* and other *Bacillus* related species) and genome size ranges from 3.7 to 6.4 Mb [10], [11]. *Bacillus* sp. strain MHSD28 genome size and G + C% content is within the range of most sequenced genomes of *Bacillus cereus* species [12], [13]. *Bacillus* sp. strain MHSD28 has 5792 total genes of which 5701 are protein coding sequences (CDSs),7 are rRNA genes with 3 operons (4 5S,1 16S and 2 23S), 79 code for tRNA genes, 5 are noncoding RNA (ncRNA) and 192 are pseudogenes. A number of genes associated with plant growth promotion activities were identified and these include siderophore production, nutrition utilization such as (nitrogen, magnesium, phosphate and potassium), growth promoting hormones [Indole-3-acetic acid (IAA)] and stress response (Table 1, Supplementary Data). Similar genes were previously identified in an endophyte *B. flexus* KLBMP 4341 [14], *B. velezensis* LDO2 [15] and *Enterobacter* sp. J49 [16].

**Table 1:** Genome statistics of *Bacillus* sp. strain MHSD28.

Attribute	Value
Genome size (bp)	5,571,729
Largest contig (bp)	1,785,042
N50	1,474,247
G + C (%)	35.23
Number of contigs	43
Total genes	5792
Total protein coding genes (CDSs)	5701
tRNAs	79
rRNAs	4,1,2 (5S, 16S, 23S)
ncRNAs	5
Pseudo genes	192

**Fig. 1:**
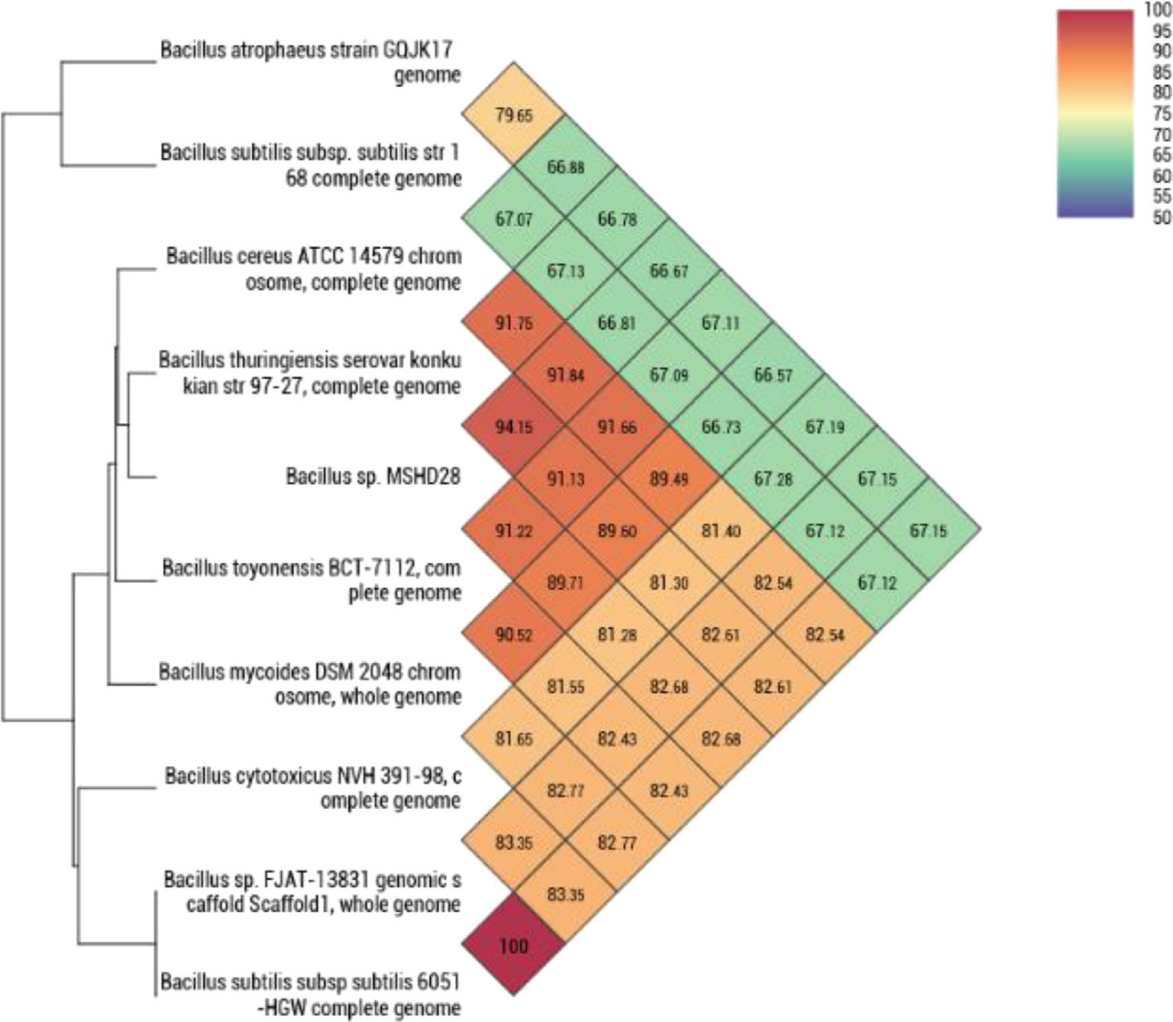
Heatmap generated with OAT software indicating the OrthoANI values of *Bacillus* sp. Strain MHSD28 and closely related *Bacillus* species.

Phylogenomic classification of MHSD28 was undertaken with the Type Strain Genome Server (TYGS), a free bioinformatics platform available under (https://tygs.dsmz) for a whole genome-based taxonomic analysis [17]. In addition, the Orthologous Average Nucleotide Identity Tool software (OAT) was used to determine the OrthoANI value with closely related species [18]. The TYGS results (Fig. 1, Supplementary Data) indicate that MHSD28 forms a monophyletic relationship with closely related *Bacillus* species. This was consistent with the extended 16S rRNA gene analysis (Fig. 2, Supplementary Data). Both trees had low δ values which corresponded to high branch support for the trees (Table 2, Supplementary Data). MHSD28 had a digital DNA-DNA hybridization (dDDH) of 77.4% with G + C% content difference of 0.03 and 70.9% with G + C% content difference of 0.04 (Table 3, Supplementary Data) with *B. tropicus* N24^T^ and *B. paranthracis* MCCC^T^, respectively. The dDDH values with both *Bacillus* species exceed the species boundary value of dDDH>70% [19]. Fig. 1 shows that strain MHSD28 exhibited OrthoANI values of 94.15% with *B. thurengiensis serovar konku*^T^, 91.84% with *B. cereus* ATCC^T^ and 91.22% with *B. toyonensis* BCT-7112^T^ all of them which are below the species boundary value (ANI, >95–96%) [18]. Phylogenomic analysis distinguishes strain MHSD28 from its closest neighbours and represents a prospective novel species of *Bacillus.* This potential new *Bacillus* species is now in the process of being described using genomic data substantiated with phenotypic and phylogenetic properties.

## 2 Experimental design, materials and methods

### 2.1 Bacterial isolation

*Bacillus* sp. strain MHSD28 was isolated from sterilized leaves of medicinal plant *Dicoma anomala* using the method described by Patle et al. [20], with some modifications. Briefly, immediately after plant material collection, in the lab, plant leaves were washed with running tap water followed by a sequential sterilization with 70% ethanol for 5 minutes, a rinse with distilled water, soak in 2% sodium hypochlorite for 3 minutes, sterile distilled water wash 3 times and the last wash plated on nutrient agar plates as control. Sterile leaves were crushed using mortar and pestle macerated with phosphate buffer (8g NaCl, 0.2 g KCl, 1.44g Na_2_HPO_4_ and KH_2_PO_4_, pH 7.4), aseptically streaked on nutrient agar plates and incubated at 30 °C for 48 hours. The plates were monitored for growth daily, grown colonies were sub-cultured several times on fresh media, preserved in 30% glycerol stock solution and stored at −80 °C for future use.

### 2.2 DNA extraction and genome sequencing

*Bacillus* sp. strain MHSD28 was cultured aerobically on nutrient agar plates at 28 °C for 24–48 hours. Extraction of genomic DNA was performed using the Zymo Research Fungal/Bacterial DNA MiniPrep Kit as per manufacturer's instructions. The quality of the DNA was assessed with a Nanodrop spectrophotometer determining A_260/280_ ratio. The DNA was sent to a commercial service provider, Agricultural Research Council (ARC), Onderstepoort in South Africa for sequencing. Illumina libraries were generated using NEBNextUltra™ II DNA library preparation kit for Illumina and paired-end (2 × 300 bp) sequenced using Illumina MiSeq instrument v3.

### 2.3 Genome assembly and annotation

All pre-annotation analysis was performed on Galaxy (www.usegalaxy.org) [21]. Quality of raw sequence data was assessed by FastQC (version 0.72). *De novo* assembly was performed using Unicycler (version 0.4.6.0) and the assembly assessed with Quast (version 0.4.6.3). The final genome assembly was annotated through the NCBI PGAAP [9].

### 2.4 Phylogenomic classification

The genome sequence data was uploaded on the Type Strain Genome Server (TYGS) (https://tygs.dsmz.de), for a whole genome-based taxonomic analysis with other validly published type strains [17]. The average nucleotide identity with closely related species was determined using the Orthologous Average Nucleotide Identity Software Tool (OAT) [18].
