# Supplementary material for: Data on draft genome sequence of Bacillus sp. strain MHSD28, a bacterial endophyte isolated from Dicoma anomala
Source: Data Brief. 2019 Sep 19;26:104524. doi: 10.1016/j.dib.2019.104524 (PMC6811918; doi:10.1016/j.dib.2019.104524)
Supplement: Multimedia component 1 [file mmc1.docx]

**Draft genome sequence of *Bacillus sp*. strain MHSD28, a bacterial endophyte isolated from *Dicoma anomala***

**Sephokoane Cindy Makuwa, Mahloro Hope Serepa-Dlamini***

Department of Biotechnology and Food Technology, University of Johannesburg, Doornfontein 2094, Johannesburg, South Africa

Contact email: [hopes@uj.ac.za](mailto:hopes@uj.ac.za)

**Table 1**

*Bacillus* sp. strain MHSD28 genes involved in plant growth promotion.

| **Function** | **Gene** | **Gene product** |
| --- | --- | --- |
| Siderophore synthesis | *dhbC*  *IucA/IucC* | Isochorismate synthase  Siderophore biosynthesis protein  Iron-siderophore ABC transporter substrate-binding protein  Iron ABC transporter permease |
| IAA synthesis | *trpA*  *trpB*  *trpC*  *kynA* | Tryptophan synthase subunit alpha  Tryptophan synthase subunit beta  Indole-3-glycerol phosphate synthase  Tryptophan 2,3-dioxygenase  GNAT family N-acetyltransferase  tryptophan-tRNA ligase protein |
| Nitrogen utilization | *mogA/moaB*  *moaA* | Molybdenum cofactor biosynthesis protein  Molybdenum cofactor biosynthesis protein  Molybdenum cofactor guanylyltransferase  Carbon-nitrogen family hydrolase  Nitrate reductase  Ammonium transporter |
| Magnesium utilization | *corA*  *mgtA* | Magnesium/cobalt transporter protein  Magnesium-translocating P-type ATPase |
| Stress response | *glsB/ yeaQ*  */ ymgE*  *Asp23/Gls24* | Stress response membrane protein  Envelope stress response protein  BA3454 family stress response protein |
| Potassium utilization | *kdpA*  *kdpB*  *trkH*  *trkA*  *ktr* | Potassium-transporting ATPase subunit  Potassium-transporting ATPase subunit  Potassium transporter  Potassium uptake protein  System potassium transporter B |
| Phosphate solubilization | *phoX*  *pstA*  *pstB*  *pstC*  *pstS* | Phosphate ABC transporter substrate-binding protein  Phosphate ABC transporter permease  Phosphate ABC transporter ATP-binding protein  Phosphate ABC transporter permease subunit  Phosphate ABC transporter substrate-binding protein  Glucose 1-dehydrogenase (gcd) |

**1 2 3 4 5 6 7 8**


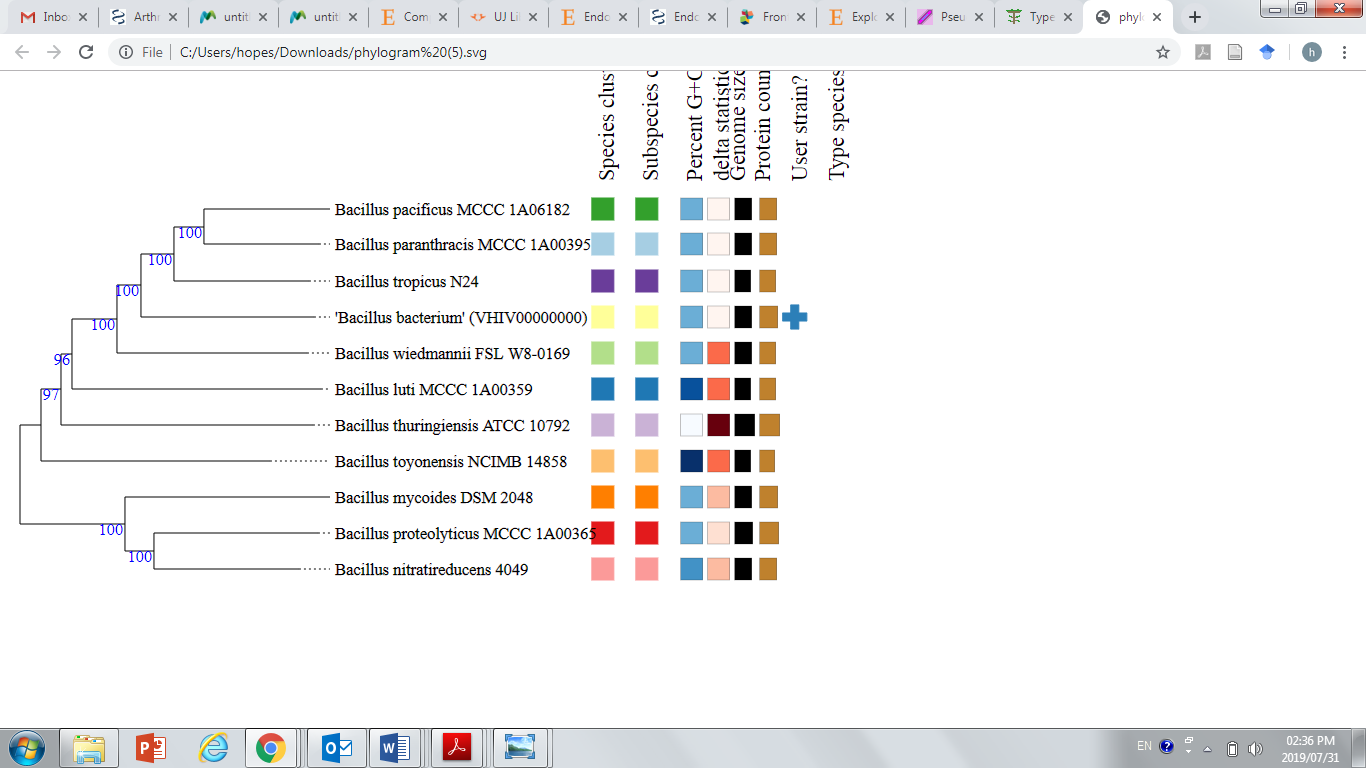


1. Species clusters
2. Subspecies clusters
3. Percent G+C (34.8-35.6%)
4. Delta statistics (0.1-0.3)
5. Genome size (5025419-6234842)
6. Protein count (4942-6243)
7. User strain [*Bacillus* sp. strain MHSD28 (VHIV00000000)]
8. Type species

**Figure 1.** Genome-Blast Distance Phylogeny (GBDP) tree inferred with FastME 2.1.6.1, GBDP distances calculated from genome sequences based on whole genome sequences, showing *Bacillus* sp. strain MHSD28 with closely related Bacillus species. GBDP pseudo-bootstrap support values from 100 replications, with an average branch support of 99.1 % are shown on branch points. The tree was rooted at the midpoint. Number 1-8 indicate species clusters, sub-species clusters, Percent G+C, Delta statistics, Genome size, Protein count, user strains and type strain respectively.


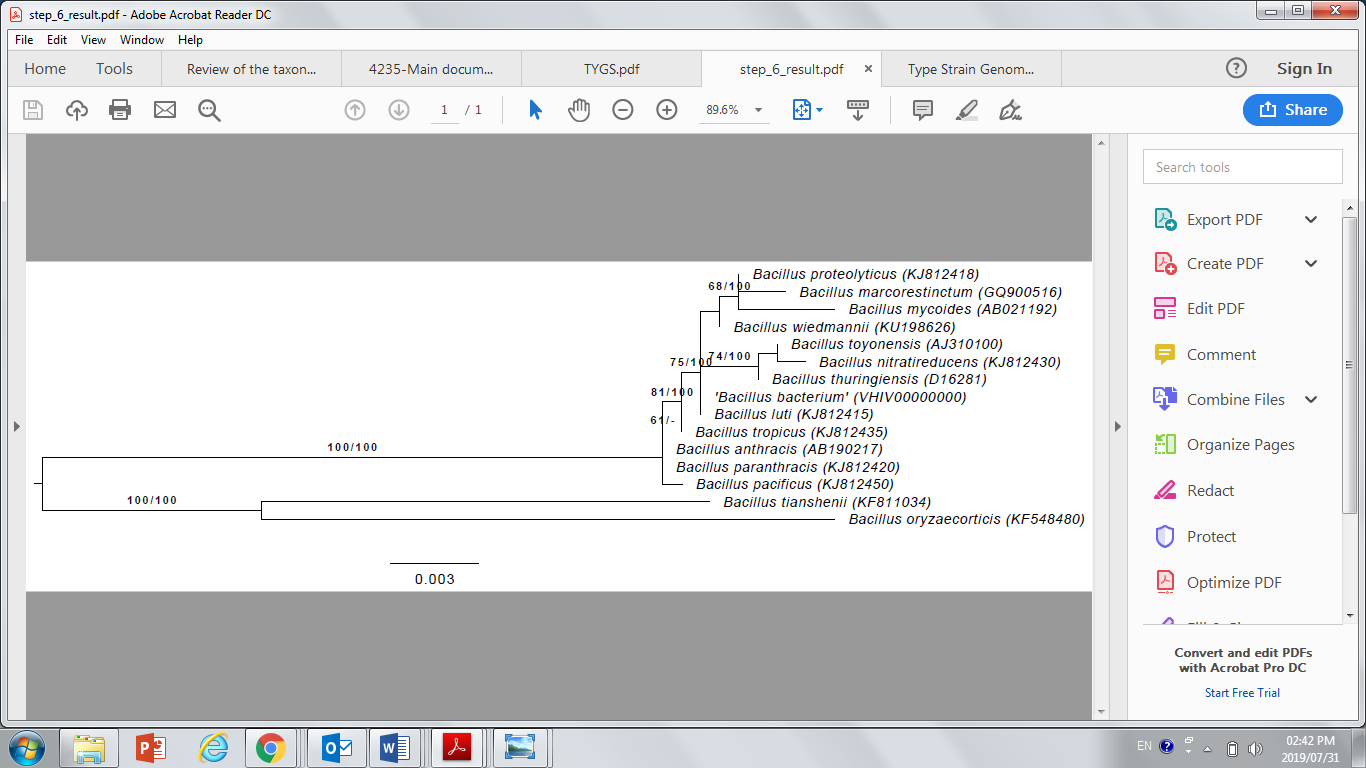


**Figure 2.** Maximum likelihood (ML) tree showing the position of *Bacillus* sp. strain MHSD28 with closely related *Bacillus* species based on 16S rDNA gene sequences inferred under the GTR+GAMMA model and rooted by midpoint-rooting. The ML (left) and maximum parsimony (MP) bootstrapping (right) branch support values (>60%) are displayed on branch points, with species accession numbers indicated in brackets. Bar 0.003substitution per site.

**Table 2**

Phylogeny statistics of the *Bacillus* sp. strain MHSD28 (VHIV00000000) and other closely related type strains.

| **Figure** | **No. strains** | **Average branch support** | **δ statistics** |
| --- | --- | --- | --- |
| **Figure 1:** GBDP tree (whole-genome sequence-based) | 11 | 99.1 % | 0.157 |
| **Figure 2:** GBDP tree (16S rDNA gene sequence-based) | 11 | 48.4 % | 0.235 |

**Table 3**

Pairwise comparisons of *Bacillus* sp. strain MHSD28 (VHIV00000000) vs. type strain genomes

| **Query strain** | **Subject strain** | **dDDH (d0, in %)** | **C.I. (d0, in %)** | **dDDH (d4, in %)** | **C.I. (d4, in %)** | **dDDH (d6, in %)** | **C.I. (d6, in %)** | **G+C content difference (in %)** |
| --- | --- | --- | --- | --- | --- | --- | --- | --- |
| 'Bacillus bacterium' (VHIV00000000) | *Bacillus tropicus* N24 | 79.9 | [76.0 - 83.3] | 55.9 | [53.1 - 58.6] | 77.4 | [74.0 - 80.5] | 0.03 |
| 'Bacillus bacterium' (VHIV00000000) | *Bacillus paranthracis* MCCC 1A00395 | 72.7 | [68.7 - 76.3] | 54.5 | [51.7 - 57.2] | 70.9 | [67.5 - 74.2] | 0.04 |
| 'Bacillus bacterium' (VHIV00000000) | *Bacillus pacificus* MCCC 1A06182 | 70.4 | [66.5 - 74.0] | 53.8 | [51.1 - 56.5] | 68.8 | [65.4 - 72.1] | 0.02 |
| 'Bacillus bacterium' (VHIV00000000) | *Bacillus wiedmannii* FSL W8-0169 | 69.9 | [66.0 - 73.6] | 52 | [49.3 - 54.7] | 67.9 | [64.5 - 71.1] | 0.03 |
| 'Bacillus bacterium' (VHIV00000000) | *Bacillus luti MCCC* 1A00359 | 66 | [62.2 - 69.7] | 44.7 | [42.1 - 47.2] | 62.3 | [59.0 - 65.5] | 0.22 |
| 'Bacillus bacterium' (VHIV00000000) | *Bacillus thuringiensis* ATCC 10792 | 62.1 | [58.3 - 65.7] | 44.7 | [42.1 - 47.3] | 59.1 | [55.8 - 62.2] | 0.4 |
| 'Bacillus bacterium' (VHIV00000000) | *Bacillus toyonensis* NCIMB 14858 | 63.8 | [60.1 - 67.5] | 43.3 | [40.7 - 45.8] | 60 | [56.8 - 63.2] | 0.33 |
| 'Bacillus bacterium' (VHIV00000000) | *Bacillus nitratireducens* 4049 | 62.7 | [58.9 - 66.3] | 40 | [37.6 - 42.6] | 57.9 | [54.7 - 61.0] | 0.07 |
| 'Bacillus bacterium' (VHIV00000000) | *Bacillus proteolyticus* MCCC 1A00365 | 58.5 | [54.9 - 62.1] | 39.7 | [37.2 - 42.2] | 54.5 | [51.3 - 57.6] | 0.08 |
| 'Bacillus bacterium' (VHIV00000000) | *Bacillus mycoides* DSM 2048 | 59.1 | [55.5 - 62.7] | 38.5 | [36.1 - 41.0] | 54.5 | [51.4 - 57.6] | 0.02 |
